# Structural Properties of MHC Class II Ligands, Implications for the Prediction of MHC Class II Epitopes

**DOI:** 10.1371/journal.pone.0015877

**Published:** 2010-12-30

**Authors:** Kasper Winther Jørgensen, Søren Buus, Morten Nielsen

**Affiliations:** 1 Department of Systems Biology, Center for Biological Sequence Analysis, Technical University of Denmark, Lyngby, Denmark; 2 Laboratory of Experimental Immunology, Faculty of Health Science, University of Copenhagen, Copenhagen, Denmark; University of Queensland, Australia

## Abstract

Major Histocompatibility class II (MHC-II) molecules sample peptides from the extracellular space allowing the immune system to detect the presence of foreign microbes from this compartment. Prediction of MHC class II ligands is complicated by the open binding cleft of the MHC class II molecule, allowing binding of peptides extending out of the binding groove. Furthermore, only a few HLA-DR alleles have been characterized with a sufficient number of peptides (100–200 peptides per allele) to derive accurate description of their binding motif. Little work has been performed characterizing structural properties of MHC class II ligands. Here, we perform one such large-scale analysis. A large set of SYFPEITHI MHC class II ligands covering more than 20 different HLA-DR molecules was analyzed in terms of their secondary structure and surface exposure characteristics in the context of the native structure of the corresponding source protein. We demonstrated that MHC class II ligands are significantly more exposed and have significantly more coil content than other peptides in the same protein with similar predicted binding affinity. We next exploited this observation to derive an improved prediction method for MHC class II ligands by integrating prediction of MHC- peptide binding with prediction of surface exposure and protein secondary structure. This combined prediction method was shown to significantly outperform the state-of-the-art MHC class II peptide binding prediction method when used to identify MHC class II ligands. We also tried to integrate N- and O-glycosylation in our prediction methods but this additional information was found not to improve prediction performance. In summary, these findings strongly suggest that local structural properties influence antigen processing and/or the accessibility of peptides to the MHC class II molecule.

## Introduction

Major histocompatibility complex (MHC) class II molecules orchestra essential parts of the immune system defining the onset of for instance cytotoxic T cell induced apoptosis and B cell proliferation. Identification of which peptides will bind a given MHC class II molecule is hence of pivotal interest for the understanding of a host immune response to any given pathogen. To guide this identification, several prediction methods have been developed over the last decade (see [1] and references herein). Prediction of naturally processed MHC class II binding peptides (MHC class II ligands) is not an easy task. The open binding cleft for MHC class II molecules allows peptides to extend the nona-mer binding core. This makes prediction of peptide binding more challenging for MHC class II compared to MHC class I due to the need to simultaneous predict the binding register and binding motif.

Antibodies have been demonstrated to be able to affect antigen processing either positively or negatively depending upon the specificity of the antibody and the CD4+T cell [2], [3], and the three-dimensional structure of antigens has been suggested to influence the processing and presentation of helper T-cell epitopes [4]. It therefore seems plausible that local structural properties of the source protein, even though not directly impacting the MHC class II binding, could impose a differential bias in the likelihood of a given peptide being processed and presented on the MHC class II molecule.

In this work, we seek to investigate this assumption and analyze if properties of peptides defined by the native local structure of the source protein influence their likelihood of being made available for binding to MHC class II molecules. The aspect of glycosylation is also included in the analysis. The vast majority of studies investigating the effect of glycosylation on T cell recognition is based on very limited amount of data and is hence highly anecdotal. Glycosylation of ligands in the MHC-II binding core region has been found to disfavour MHC class II binding [5]. When present in the binding core, some evidence indicates that these carbohydrate moieties play an important role in T-cell recognition [6]. Glycosylations of the flanking amino acids are found more frequently since these often will allow the T-cell receptor to get in contact with the MHC:peptide complex [7]. Here, we investigate using a large benchmark data set, whether ligands are glycosylated or not and if the glycosylation is overrepresented in MHC class II ligands compared to the background.

Two large-scale benchmark data sets were used for the analysis consisting of MHC class II ligands obtained from the SYFPEITHI database [8]. Native local protein structure properties were predicted using *NetSurfP* [9]. Binding affinities to the MHC class II molecules were predicted using *NetMHCIIpan* version 1.0 [10], and N- and O-glycosylation sites were predicted using *NetNglyc* [11] and *NetOglyc* [12]. Using these predicted features, we seek to analyze if structural properties for MHC class II ligands differ from other non-ligand peptides with equal binding affinity to the relevant MHC-II molecule. Demonstrating such a structural differential bias, we next attempt to combine the local structural information with the binding strength to the MHC-II molecule in a model with improved accuracy of prediction for MHC class II ligands.

## Results

### Ligands versus non-ligand binders

We first compared the local structural properties of the MHC-II ligands to that of the corresponding non-ligand peptides. We define a non-ligand binder as an affinity matched peptide within the ligand source protein not overlapping with the ligand peptide, where affinity matched is a predicted binding affinity (in log-transformed units) in the range ±5% of the binding affinity of the MHC-II ligand. For 87 out of 644 ligands, the criterion (±5%) did not result in any selected peptides because these ligands were predicted by *NetMHCIIpan* to be very strong binders, and no other peptides were found within the source protein with a binding affinity within the range of -5% of the reference ligand binding affinity. Requiring that peptides in the non-ligand data set were found both with stronger and weaker binding affinities compared to the reference ligand further reduced the set of ligand:non-ligand binder pairs to 459. When multiple peptides in the source protein fulfilled the above criteria for non-ligand binder, a random peptide from the non-ligand pool was selected.

Performing a pairwise comparison of local structural properties between these 459 ligands and their non-ligand affinity matched counter-part revealed that MHC class II ligands were significantly more exposed and had significantly less secondary structure element (α-helix and β-strand) compared to the non-ligands (see Table 1). The difference in binding affinity for ligands and non-ligands was found not significant (p<0.895, paired t-test).

**Table 1:** Mean and standard deviation value for ligands and non-ligands compared for the different groups.

Class	Ligand	Non-ligand	P-value
α-helix	0.231±0.276	0.285±0.322	P<0.002
β-strand	0.289±0.207	0.279±0.232	P<0.396
α+β	0.520±0.171	0.564±0.192	P<0.0002
Coil	0.480±0.076	0.437±0.192	P<0.0002
RSA	0.298±0.076	0.273±0.099	P<0.0013
1-log50k	0.404±0.173	0.404±0.173	P<0.895

P-values are obtained from a paired t-test. Class indicates the different classes/methods used in the analysis. The first groups are self-explanatory (i.e. α-helix, β-strand, α+β, and coil). RSA is the relative surface accessibility. All these values are obtained using *NetSurfP*. 1-log50k is the binding affinity in log-transformed units obtained from *NetMHCIIpan*.

To investigate if this difference in local structural properties could be contributed to a bias in the *NetsurfP* method used to predict the local structural properties, a comparative analysis was performed using the *Real-SPINE* 3.0 method for prediction of residue solvent accessibility [13]. Real-SPINE 3.0 was able to return residue solvent accessibility predictions for the source-proteins of 457 of the 459 ligand included in table 1. Using these predicted exposure values, the mean RSA for the ligand and non-ligands peptides was found to be 0.214+/− 0.048, and 0.205+/− 0.058, respectively. These values are different from the values obtained using the *NetSurfP* method (see table 1). However, the difference between the two peptides sets is statistically significant (p<0.05, paired T test). This result thus strongly suggests that the results obtained in this work are robust and not influenced by the method applied to obtain local structure predictions, as long as the method is indeed state-of-the art. Through out the remaining part of the manuscript only *NetSurfP* predictions were used.

### Training set

To investigate if these findings could be applied to improve the *in silico* identification of MHC class II ligands, a simple model was created which integrated the MHC-II binding affinity with a structural feature or surface exposure as described by Eq.(1).
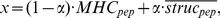
(1)where x is an MHC class II ligand likelihood score, MHC_pep_ represents the MHC binding affinity and 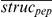 is the mean value of a structural class (i.e. α-helix, β-strand, coil or RSA) for the peptide. Brute force grid search was used to identify the value of α that gave the optimal AUC0.1 (see material and methods) performance measured on the balanced training set.

The average predicted binding affinity for ligands restricted to different HLA alleles is often very different. For instance is the average 1-log50k predicted binding value for the ligands in the training data set restricted to the alleles HLA-DRB1*0101 and HLA-DRB1*0301 0.49±0.19 and 0.19±0.14, respectively. This difference is highly statistically significant (p<0.005, t-test). Even though these differences are based purely on predicted binding affinities, the findings correspond to what has been observed for MHC class I binding, where evidence is merging suggesting that MHC class I molecules present peptide on the cell surface at different binding thresholds [14], [15]. Thus, to identify an optimal and HLA universal α-value across all the different HLA-DR alleles, a re-scaling was made for all predicted binding affinities. For every allele, the 1-log50k(IC50 nM) binding affinities were rescaled with a given percentile score. This percentile score was calculated for each specific allele using a set of 200.000 random natural 15-mer peptides. The binding affinities were hereby rescaled according to the percentile chosen. Rescaling at percentiles from 1-5 gave very similar results (data not shown). Rescaling was decided to be based on the 1-percentile (Rescaled01) since this gave a slightly improved performance compared to other percentile values when evaluated on the balanced training data.

We now use the balanced training data set to define the optimal value of α for the model defined using Eq. (1). All details of this calculation are found in Table 2. The AUC0.1 for the raw *NetMHCIIpan* method was found to be 0.293. When combined with relative surface exposure an optimal value of α_RSA_ was found to be 0.30 with an AUC0.1 performance of 0.312. This difference between the model and the *NetMHCIIpan* method is highly statistically significant (p<3.9·10^−4^). When combining the rescaled *NetMHCIIpan* predicted binding affinity with coil predictions an optimal α_coil_ value was found to be 0.20 with an AUC0.1 value of 0.317. Also, this increase in performance between the model and the raw *NetMHCIIpan* method is statistically significant (p<6.3·10^−4^). For the remaining part of the training set, the two models with α_RSA_ = 0.30, and α_coil_ = 0.20, respectively, significantly outperformed the *NetMHCIIpan* method (p<10^−7^, and p<3.5·10^−3^).

**Table 2:** Predictive performance of the model compared to *NetMHCIIpan* as measured by AUC0.1 and AUC.

RSA	*NetMHCIIpan*		Model – Rescaled01			
	AUC	AUC0.1	α	AUC	AUC0.1	P-value
Balanced training set	0.781	0.293	0.3	0.784	0.312	<0.0004
Rest of training sset	0.823	0.334	0.3	0.834	0.371	<10^−7^
Test set	0.796	0.318	0.3	0.792	0.329	<0.02

Coil	*NetMHCIIpan*		Model – Rescaled01			
	AUC	AUC0.1	α	AUC	AUC0.1	P-value
Balanced training set	0.781	0.293	0.2	0.782	0.317	<0.0005
Rest of training set	0.823	0.334	0.2	0.829	0.351	<0.004
Test set	0.796	0.318	0.2	0.795	0.318	<0.98

The balanced set was used to identify the optimal weights for RSA and coil combined with rescaled binding affinities (Rescaled01) as define by Eq. (1). The optimal α-values for each model are given in the table. P-values are given by paired t-tests when comparing AUC0.1 of the model to the *NetMHCIIpan* method. Rest of training set refers to the training set, and test to the 697 ligands in the test set.

To estimate the robustness of the model parameter α, 5-fold cross validation on the balanced training set was performed. In the cross-validation 4/5 of the data were applied to estimate the optimal model parameter α, and the remaining 1/5 of the data was next used as test set and predicted using this optimal value of α. This procedure was repeated five times ensuring that all data points form part of the test set exactly one time. For RSA, the AUC0.1 value obtained using five-fold cross-validation was 0.309, and the average optimal α for the 5 cross-validations was 0.330±0.045, and similarly was the AUC0.1 value 0.303 for the model using coil with an average optimal α for the 5 cross-validations 0.220±0.027. These low values on the standard deviation of the α values indicate that the model is robust and that is does not suffer from noticeable overfitting.

Next, a model combining MHC binding, RSA and coil was investigated. The model was defined as described by Eq. (2).
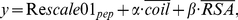
(2)where Rescale01_pep_ is the rescaled binding affinity of the peptide to the given HLA allele, 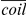 is the average predicted coil score of the peptide, 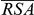 is the average predicted relative exposure score of the peptide, and α and β are relative adjustable weights. Brute force grid search was used again to define weights on RSA and coil that optimized the AUC0.1 value on the balanced training data. The best performing model (2) was achieved with α = 0.1 and β = 0.5 given an AUC0.1 of 0.351. This value is significantly higher than the AUC0.1 value of 0.315 obtained by the *NetMHCIIpan* (p<2.0·10^−9^). However, the improvement was not significant for the model combining RSA and coil compared the model with RSA alone (p<0.345). This more complex model was therefore not investigated further.

### Test set

Next, the model define by Eq. (1) was investigated using the 697 ligands in the test set. The optimal α-value of 0.30 for RSA was chosen based on the previous results. The performance measured by AUC0.1 was increased from 0.318 (*NetMHCIIpan*) to 0.329 (p<0.018) for MHC binding combined with RSA. For MHC binding combined with coil, the α-value of 0.20 gave a slight decrease in the performance from 0.318 to 0.318 (p<0.978). For details see Table 2.

The model combining MHC affinity with RSA thus consistently and significantly improved the predictive performance compared to MHC binding alone on all benchmark data sets, thus supporting the consistency of the model. In contrast to this, did the model with coil combined with MHC affinity not improve the predictive performance above what is obtained using MHC binding alone when evaluated on the test set.

### Glycosylation

The previous analysis demonstrated significant differences in local structural properties between ligands and affinity matched non-ligands. Here, we apply a similar approach to investigate if differences existed between ligands and affinity matched non-ligands with respect to glycosylation. The comparison between the 459 ligands and non-ligands was used again to identify trends regarding glycosylation. For all the corresponding source proteins N- and O-glycosylation were predicted, and the number of predicted glycosylation within ligands/non-ligands was calculated. Out of the 459 ligands:non-ligand pairs, 27 ligands were predicted to be glycosylated. For the non-ligands this number was 50. The ligands were thus predicted to be significantly (p<0.012, binomial test) less glycosylated than the non-ligands (see Table 3).

**Table 3:** Comparison of glycosylation between ligand and non-ligands.

Class	Ligand	Non-ligand	P-value
l-log50k	0.404±0.173	0.404±0.173	<0.895
N-glyc	20	40	<0.015
O-glyc	7	10	<0.63

All the 459 ligands with corresponding non-ligands were analyzed in respect to N- and O-glycosylation. Ligands and non-ligands were defined as described in the text. P-values are based on binomial tests, with a hypothesized proportion of 0.5.

The full training set and test set were also analyzed according to the glycosylation sites for both N- and O-glycosylation. Out of the 644 ligands in the training set, 6.2% were predicted glycosylated (25 N- and 15 O-glycosylated). Out of the 697 ligands in the test set, 9.6% were predicted glycosylated (36, N- and 31 O-glycosylated). No ligands from the training and the test set were predicted both N- and O-glycosylated. Taking all peptides within the source proteins as the background, it was found that the predicted background frequency for glycosylation for the training set was 9.11% (29654 peptides out of 325276) and for the test set 10.7% (47958 peptides out of 447453). For both data sets, the background frequency of glycosylation was thus slightly higher than what was found to the corresponding ligands, suggesting the MHC class II ligand presentation could be interfered by glycosylations.

An attempt was made to improve the prediction of MHC class II ligands combining *NetMHCIIpan* binding affinity and glycosylation. Several models were investigated. Even though we showed above that the ligands are less glycosylated than affinity matched non-ligands, no model was found that consistently improved the predictive performance on all three data sets.

## Discussion

Characterizing and identifying peptides that bind MHC class II molecules and elicit an immunogenic response is critical for the understanding of host-pathogen immune system interactions and in the selection of candidate peptides in vaccine research. The process of identifying such peptides is however a highly resource intensive and difficult task.

During the recent decade several *in silico* prediction algorithms have therefore been developed aiming at guiding and cost-reducing the task of identifying T cell epitopes. While the accuracy for prediction of MHC class I restricted epitopes/ligands has reached a level where only few percent of the predictions turn out to be false [13], the situation for MHC class II is different. Here the algorithms, even though recently achieving improved predictive performances, maintain a relative low accuracy when it comes to MHC epitope/ligand discovery [1].

The majority of the methods developed for prediction of MHC class I restricted ligands and to our knowledge all methods developed for prediction of MHC class II restricted ligands focus on prediction of the peptide:MHC binding event alone. The classical pathway for MHC class II ligand presentation involves uptake of protein or protein fragments through endocytosis or phagocytosis by antigen presenting cells (APC). Antibodies can enhance specific source antigen uptake and presentation to CD4+T cells by orders of magnitude (reviewed in [16], [17], [18]). Once the source antigen has been internalized and processed, one would a priori expect that any peptide derived from this source antigen would be offered to MHC class II and could be presented. However, antibodies have been demonstrated to be able to affect antigen processing either positively or negatively depending upon the specificity of the antibody and the CD4+T cell. This T-B cell reciprocity has been suggested to be the result of antibodies affecting the outcome of antigen processing in ways that are not easy to predict. The effect of an antibody that binds to and protects the bound epitope itself could be to protect the determinant (enhanced presentation), whereas the effect of an antibody that binds to and protects a site that needs to be processed in order to liberate the epitope could lead to inhibition of processing (reduced presentation). Both effects have been demonstrated in the literature [2], [3].

Since the majority of B cell epitopes are characterized by a structural signature in that they tend to protrude at the protein surface and be highly exposed [19], [20], we postulated that upon uptake by the antigen presenting cell, the local structural properties of an epitope, in the context of the native of the structure source protein, could impose a differential bias in the likelihood of a given peptide epitope being appropriately processed and presented. Moreover has earlier work on antigen processing and presentation suggested that antigen three-dimensional structure might influence the processing and presentation of helper T-cell epitopes [2], [3], [4].

Here, we further investigated this hypothesis, and analyze to what extent the local structural properties of an epitope, in the context of the native structure of the source protein, can impose a differential bias in the likelihood of a given peptide epitope being appropriately processed and presented. We investigated this hypothesis on a large set of MHC class II ligands from the SYFPEITHI database. Using the state-of-the-art MHC class II binding predictions, *NetMHCIIpan*, combined with prediction of local protein structure by *NetSurfP*, our analysis revealed that HLA class II ligands are significantly more exposed and have significantly less local secondary structure elements compared to affinity matched non-ligand peptides within the same source protein. Since the source protein is internalized as a unit and then processed, our comparison neutralizes the effect upon antigen uptake, which otherwise would confound the issue of the effect upon antigen processing. We suggest that our observation could be the result of antibody-mediated determinant protection and that this effect dominates over antibody-mediated inhibition of processing. This interpretation of our results would agree with recent finding by Sette et al. [21], that in a vaccinia model demonstrated that CD4+T cells and neutralizing antibodies target the same protein antigen. Alternatively, the observed structural bias in MHC ligands could stem from the digestion of the antigen in the endosomes, since it is possible that surface exposed peptide fragments are more effectively be released from the antigen protein structure compared to fragments in the more structurally stable hydrophobic protein core.

Next, we proposed a method for prediction of MHC ligands combining MHC class II binding predictions with local structure prediction, and demonstrated that this method consistently in three benchmark studies significantly improved the prediction accuracy of a method based on MHC binding alone.

It is important to stress that our observations should not be taken as an indication for MHC class II ligands being absent form the protein core. Our observations merely demonstrate that a differential bias exists and that exposed peptide fragments are more likely presented by MHC class II molecules compared to affinity matched buried peptides.

We also investigated if glycosylations could have a potential influence on the likelihood of peptides being presented on MHC class II molecules, and demonstrated that the MHC class II ligands are significantly less glycosylated compared to affinity matched non-ligands. All the ligands used in this work were obtained from the SYFPEITHI database. The ligand data in this database might have a certain bias with respect to glycosylation due to the experimental procedure in which they are defined (Stefan Stevanovic, personal communication). Thus the conclusions regarding glycosylation should be read with some caution.

In conclusion, this work has shown strong evidence that local structural properties of proteins will significantly bias processing and presentation of the corresponding peptides, and that highly exposed peptides will have a higher likelihood of being presented on the cell surface in complex with MHC class II molecules compared to other affinity matches but less exposed peptides. We further demonstrate how this finding in a simple way can be applied to significantly improve the predictions of MHC class II ligands by combining predicted surface exposure with state-of-the-art prediction methods for MHC class II binding.

## Materials and Methods

### Data

The analysis was run on two data sets; a training set and a test set. The training data set contained 644 unique ligands covering 22 HLA-DR molecules and the test set contained 697 unique ligands covering 28 HLA-DR molecules. All ligands were obtained from the SYFPEITHI database [8] and no ligands were common to both data sets. The source protein for every ligand was searched in the Uniprot database [22]. If more than one hit existed, the longest protein was chosen. The two data sets are summarized in Table 4.

**Table 4:** The MHC class II ligands distribution across alleles for the two data sets.

Allele	Training set	Test set	Allele	Training set	Test set
DRB1*0101	13	47	DRB1*1104	7	2
DRB1*0102	5	1	DRB1*1201	8	6
DRB1*0301	20	89	DRB1*1301	14	12
DRB1*0401	365	154	DRB1*1302	14	9
DRB1*0402	33	4	DRB1*1401	3	7
DRB1*0403	-	1	DRB1*1501	2	21
DRB1*0404	43	4	DRB1*1502	-	3
DRB1*0405	26	10	DRB1*1601	-	2
DRB1*0701	23	27	DRB3*0101	-	3
DRB1*0801	33	7	DRB3*0202	3	-
DRB1*0802	-	1	DRB3*0301	3	2
DRB1*0803	-	1	DRB4*0101	1	5
DRB1*0901	4	2	DRB4*0103	-	2
DRB1*1001	1	241	DRB5*0101	7	14
DRB1*1101	16	20	Total	644	697

The training set was analyzed thoroughly according to the distribution of alleles. The allele HLA- DRB1*0401 constituted more than half of the data set alone - 365 ligands out of 644 ligands. To reduce the allelic bias imposed by the uneven distribution of ligands per allele in the training set, a balanced subset of the training set consisting of no more than 30 ligands per allele was created. This balanced training data set consisted of 290 ligands.

### MHC-II ligands and non-ligand MHC-II binding peptides

In the data set of MHC-II ligands, we only have access to information on which peptides in the given source-protein are MHC-II ligands. To define a negative set of non-ligand peptide, we take an approach described earlier in for validation of prediction methods for both MHC class I and class II (see for instance [15], [23]), and construct the negative set from all other peptides in the source-protein not equal to a known MHC ligand. The rational behind this approach is that the MHC molecule is very specific and will only bind and present a very small fraction of the source protein peptides [24], [25]. Assuming that only one peptide is presented from each source-protein is clearly an over-simplification, and it is very likely that some of the non-ligands might be falsely classified. However, such erroneous classifications will not invalidate the approach but merely introduce noise lowering the overall predictive performance of the methods. If we despite this potential noise observe a significant signal in our analysis, then the signal would become even stronger in the ideal (but impossible) situation where also the negative data set was experimentally validated.

### Methods

The source protein was cut into overlapping peptides of a length equal to the corresponding MHC-II ligand, and the pan-specific prediction server, *NetMHCIIpan* version 1.0, which computes the binding affinity in nM units and reports both this value and a log50k transformed (i.e. 1-log50k(IC50 nM)) binding value [10], was applied to predict for each peptide the binding affinity to the MHC-II molecule in question. Local protein structural features for each of the source protein peptides were predicted using the *NetSurfP* method [9]. This method predicts for each amino acid the relative surface accessibility (RSA), as well as probabilities the residue being in an α-helix, β-stand, or coil secondary structure element. The output from *NetSurfP* was processed to generate mean values for the secondary structure elements: α-helix, β-strand, coil and RSA for each peptide in the source protein. Thus every peptide was coupled to a predicted MHC class II binding affinity as well as mean values for α-helix, β-strand, coil and RSA. The glycosylation sites were predicted with *NetNglyc* and *NetOglyc* with default parameter settings [11], [12].

### Performance measures

The predictive performance was measured in terms of the area under the ROC curve (AUC). For each ligand the corresponding source protein was split into overlapping peptide sequences of the length of the ligand. All peptides except the annotated HLA ligand were taken as negatives. This is a very stringent assumption since for instance suboptimal peptides sharing the ligand binding-core are counted as negatives even though they could be presented on the HLA molecule. Thus, this setup is likely to underestimate the predictive performance, but the effect should be equal for all methods compared in the benchmark. AUC values were calculated for each protein-HLA ligand pair and the overall predictive performance was next measured as the average AUC value per protein-HLA ligand pair over the data set. Since the balance between positive and negative peptides in the data sets is highly skewed with the majority of the peptide being negative, the AUC measure might not be optimal if a prediction method is required to have a high specificity in order to lower the false positive rate for subsequent experimental validation. In such situations, it is beneficial to use only the high specificity part of the ROC curve to calculate a fractional AUC value [15]. Here, the AUC0.1 (AUC integrated up to a specificity of 0.9) value was used.

### Statistical tests

Paired t-tests and binomial tests were used to access differences between ligands and non-ligands as well as differences between *NetMHCIIpan* and the new method. P-values less than 0.05 were considered statistical significant.
